# She adapts to her student: An expert pragmatic speaker tailoring her referring expressions to the Layman listener

**DOI:** 10.3389/frai.2023.1017204

**Published:** 2023-03-09

**Authors:** Claudio Greco, Diksha Bagade, Dieu-Thu Le, Raffaella Bernardi

**Affiliations:** ^1^CIMeC, University of Trento, Rovereto, TN, Italy; ^2^Amazon Alexa AI, Berlin, Germany; ^3^DISI, University of Trento, Povo, TN, Italy

**Keywords:** neural conversational models, adaptive model, grounded reference game, knowledge disparity, image captioning generation

## Abstract

Communication is a dynamic process through which interlocutors adapt to each other. In the development of conversational agents, this core aspect has been put aside for several years since the main challenge was to obtain conversational neural models able to produce utterances and dialogues that at least at the surface level are human-like. Now that this milestone has been achieved, the importance of paying attention to the dynamic and adaptive interactive aspects of language has been advocated in several position papers. In this paper, we focus on how a Speaker adapts to an interlocutor with different background knowledge. Our models undergo a pre-training phase, through which they acquire grounded knowledge by learning to describe an image, and an adaptive phase through which a Speaker and a Listener play a repeated reference game. Using a similar setting, previous studies focus on how conversational models create new conventions; we are interested, instead, in studying whether the Speaker learns from the Listener's mistakes to adapt to his background knowledge. We evaluate models based on Rational Speech Act (RSA), a likelihood loss, and a combination of the two. We show that RSA could indeed work as a backbone to drive the Speaker toward the Listener: in the combined model, apart from the improved Listener's accuracy, the language generated by the Speaker features the changes that signal adaptation to the Listener's background knowledge. Specifically, captions to unknown object categories contain more adjectives and less direct reference to the unknown objects.

## 1. Introduction

Thanks to the research on multi-task learning and transferability (Ruder, 2019), the current trend in natural language understanding is to build “universal models”: models pre-trained on several tasks and fine-tuned on a downstream task to which the acquired conceptual knowledge and skills are transferred. These steps are very important contributions to AI, but universal models do not model the human intelligence diversity. Current conversational models based on such universal encoders have shown astonishing results. However, among humans, there is no such thing as a “universal model” which all humans share. We might share the salient aspect of our neural architecture but we differ on the tasks we experience through life, on the conceptual knowledge we acquire, and on our expertise or cultural background. Therefore, we believe it is important to work with conversational models which have different prior experiences.

To communicate effectively, speakers and listeners must coordinate their use and interpretation of language. For instance, which referring expressions they use to refer to entities or events depends on the information they share (Clark and Marshall, 1981; Horton and Keysar, 1996; Brennan et al., 2010). As discussed by Garrod and Pickering (2004), the success of conversations depends on the extent to which the Speaker and the Listener have a similar representation of the elements they speak about (e.g., they should refer to the same individual when using the same name). Moreover, humans are able to understand the goals and intentions of the others, as well as the others' beliefs, doubts, etc. They are said to have a “Theory of Mind” (ToM) (Premack and Woodruff, 1978). In the last year, several works have been published within the computational linguistic community pointing back to the importance of developing models with a ToM (Bisk et al., 2020; Bara et al., 2021; Zhu et al., 2021). An intelligent conversational system should be aware of what it knows and be able to quickly revise its knowledge when useful counter-evidence to its beliefs is met. Furthermore, it should be able to use language differently based on the interlocutor's knowledge, and it should be able to make predictions about its interlocutor's expectations and tailor its communication to them. In this paper, we contribute to a such ambitious long-term goal by focusing on the audience-design aspect of ToM.

The human ability to tailor language use to the interlocutor has been long and deeply investigated through psycholinguistic studies. Human partners were set to play iterative reference games: they were given a set of objects among which the Speaker was privately assigned a target to describe and the Listener had to identify it within the given contrast set (Krauss and Weinheimer, 1964; Clark and Wilkes-Gibbs, 1986; Gann and Barr, 2014; Hawkins et al., 2020b; Loy and Smith, 2021). Various theoretical models of language production have been proposed to account for the audience-design process that emerged through such experiments (e.g., Horton and Keysar, 1996; Brennan et al., 2010; Gann and Barr, 2014). More recently, human alignment through conversation has also attracted the attention of neuroscientists (Hasson et al., 2012; Kuhlen et al., 2017). In the development of conversational agents, this core aspect was on the agenda of the pre-neural era (e.g., Janarthanam and Lemon, 2010), but it has been put aside for several years since the main challenge has been to obtain conversational neural models able to produce utterances and dialogues that are human-like at least at the surface level. Now that this milestone has been achieved, the importance of paying attention to the dynamic and adaptive interactive aspect of language has been advocated in several position papers (Bisk et al., 2020; Benotti and Blackburn, 2021; Chandu et al., 2021). Inspired by the seminal work on multi-agent settings carried out within the language emergence research line (Lazaridou et al., 2017, 2020), the community has also been challenged to consider multi-agent interactions in which a speaker has to interact and adapt to a population of listeners with different visual (Corona et al., 2019), or different linguistic (Zhu et al., 2021) abilities. In this paper, we focus on how a Speaker adapts to an interlocutor with different background knowledge. Following Hawkins et al. (2020a), our models undergo a pre-training phase, through which they acquire grounded knowledge by learning to describe an image (Image Captioning, IC), and an adaptive phase, through which a Speaker and a Listener play a repeated reference game. Hawkins et al. (2020a) study how conversational models create new conventions. Instead, we are interested in studying whether the Speaker learns from the Listener's mistakes to adapt to his background knowledge.

**Figure 1:**
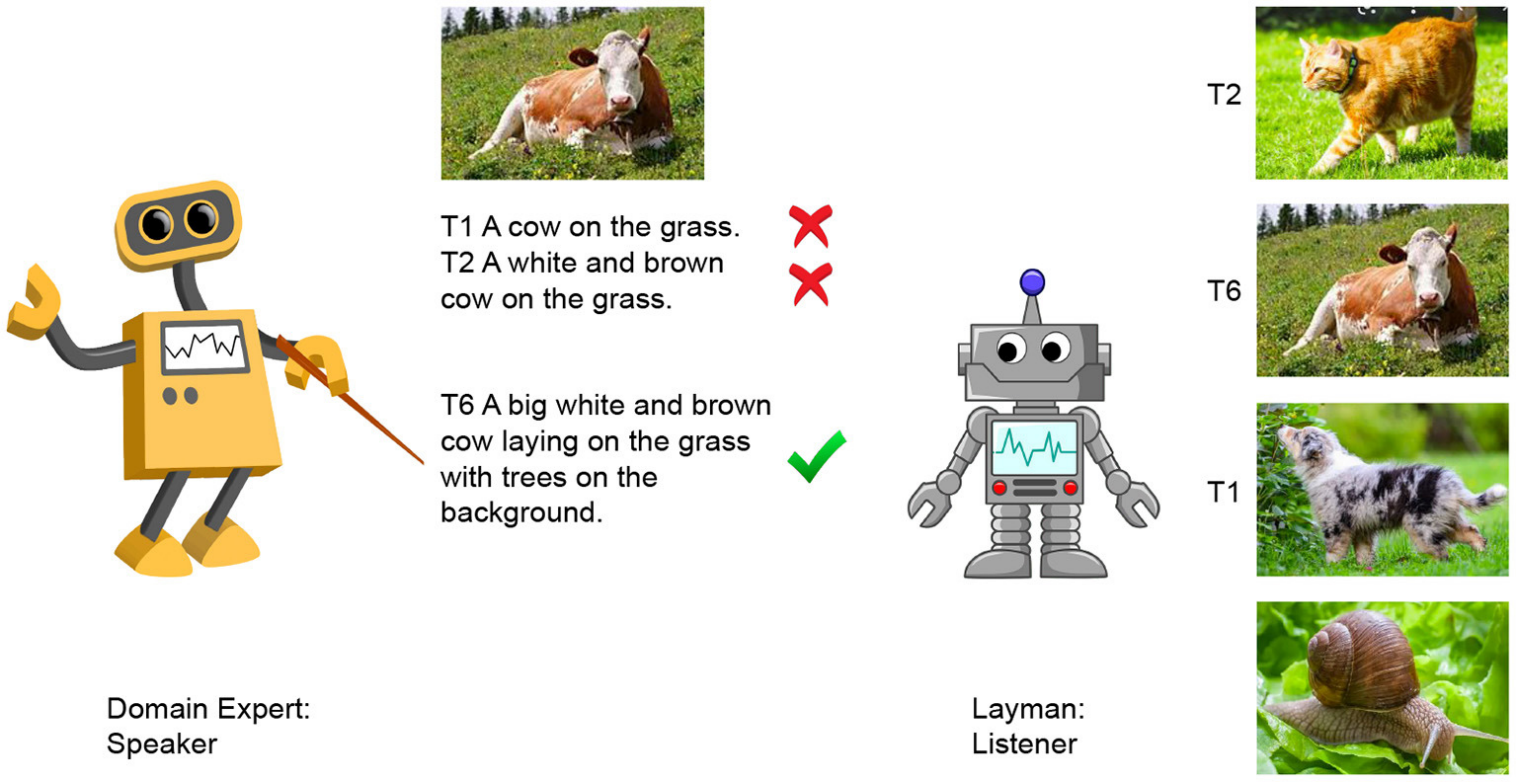
Iterative reference game between an Expert and a Layman. The Expert describes a given image, while the Layman selects one target among four within-domain candidates. Through the 10-turn game, the Expert learns to adapt her language to the Layman by learning from his mistakes.

As illustrated in Figure 1, we simulate the situation in which a domain expert interacts with a non-expert (a layman); for the communication to be successful the expert has to understand what the layman does not know and learn to adapt her language accordingly. Specifically, the Speaker is the expert who knows all concepts within a certain domain, while the Layman lacks some of them. For instance, he knows what *animal* means, since he has seen dogs and cats, but he lacks the concept of a *cow* because he has never seen one during the pre-training phase. We simulate the expert vs. layman's different backgrounds by controlling for the concepts the models see during the IC pre-training; we create contrast sets by controlling the presence of concepts unknown to the layman. In line with psycholinguistic studies (Horton and Keysar, 1996), we hypothesize that if the Speaker, through repeated interaction, learns what the Listener does not know, she will end up describing the objects belonging to unknown categories with more visual details since only naming them will not be sufficient. While writing the paper, we found Bao et al. (2022) who also tackle the issue of knowledge disparity, but, from what we understood, they focus on the Listener missing some words in her vocabulary, rather than missing the actual experience of the corresponding objects.

In order to tackle this task, we propose a model that combines the adaptation approach proposed in Hawkins et al. (2020a) with the Rational Speech Act (RSA) model (Frank and Goodman, 2012; Cohn-Gordon et al., 2018). The model is trained to describe the concepts unknown to the Listener by leveraging on the Listener's mistakes.^1^ Our main aim is to call attention on the dynamic, incremental aspect of communication and in particular on the ability of the Speaker to adapt to its interlocutor's knowledge. We show that the RSA framework could drive the Speaker to align with her Listener and we believe more work needs to be done in order to obtain robust models that learn in a more robust way what they have achieved thanks to the RSA support through interactive interactions.

## 2. Related work

Our work combines two research lines: the use of iterative games to simulate repeated communications through which speakers adapt to their partners, and the modeling of pragmatic speakers that take their partners' reasoning into account when interacting with them.

### 2.1. Adaptation

Within cognitive science, it has long been recognized that communication is a joint effort (see e.g., Grice, 1975; Clark and Wilkes-Gibbs, 1986; Clark, 1992), during which two or more interlocutors align their mental states (e.g., Brennan and Clark, 1996). It has been shown that humans employ audience design processes, in other words, they adapt their language to their interlocutor (Clark and Marshall, 1981). It has been argued that the study of the language faculty should be tightly connected with the study of the ability to represent the other's mental state (Theory of Mind) (see e.g., Brennan et al., 2010). The psycholinguistic literature is rich of interesting studies about how humans adapt their language taking into account their interlocutor's characteristics and prior experiences (global level) as well as cues that emerge as the dialogue unfolds (local level), or the interplay between these two levels. Such studies are carried out by letting subjects interact through repeated reference games (Krauss and Weinheimer, 1964; Clark and Wilkes-Gibbs, 1986; Clark, 1992; Gann and Barr, 2014). Based on these experiments several theoretical models of human adaptive ability have been proposed. As nicely summarized in Brennan et al. (2010), most of them rely on the notion of common ground, i.e., the information that the interlocutors have reason to believe is mutually shared) (Stalnaker, 1978; Clark and Marshall, 1981), while differ on how such belief impacts the audience design during the language production (see e.g., Horton and Keysar, 1996; Horton and Gerrig, 2005, 2016; Brennan et al., 2010; Gann and Barr, 2014). For instance, some models see it playing a role during the initial utterance design by the Speaker (“Initial Design”), while others assume it enters into place only later (“Monitoring and Adjustment”): if the Speaker is not under time pressure, he will take common ground into account, exploit his memory and revise the initial plan that violates it (Horton and Keysar, 1996).

In the past few years, several works have been published within the computational linguistic community pointing back to the importance of developing models with a Theory of Mind (ToM) (Bisk et al., 2020; Bara et al., 2021; Zhu et al., 2021); one of the aspects of ToM that has received more attention is the ability conversational models should acquire to adapt to the interlocutor. Adaptation has been tested considering iterative reference games (Hawkins et al., 2020a) or language navigation tasks (Zhu et al., 2021), modeled through continual learning or few shot learning paradigms. In both cases, the agents interact for *N* games through which the Speaker is expected to adapt to the Listener. In particular, Hawkins et al. (2020a) introduce a communication task along with a general continual learning framework for adapting language models during real-time interactions with other agents. The task proposed in the paper is a repeated reference game, in which the Speaker and the Listener are shown a set of images and must collaborate to refer to them. During each trial, one of the images is privately designated as the target for the Speaker, which must generate an utterance describing the target. The Listener must then guess the target based on the context and the Speaker's utterance. Both agents receive feedback on the listener's response and the identity of the target. The sequence of trials is designed so that each image repeatedly appears as the target, allowing us to track the performance and the way the communication about each image changes over time. The approach described in the paper is motivated by hierarchical Bayesian approaches to task-specific adaptation and it involves combining speaker and listener information in a loss function, as well as using a regularization scheme to fine-tune model weights without overfitting. The model is implemented by combining the standard cross-entropy term (that accounts for the most likely utterance in isolation) with a contrastive term (that accounts for the most discriminative utterance) and with a KL-based regularization term (aiming at preventing catastrophic forgetting). Our paper builds on this work, by using an iterative reference game; differently from what has been done so far, we consider an Expert-Layman setting and study how the expert adapts to the layman's partial knowledge through iterative communication about the same entities or the same domain. Moreover, we also evaluate the impact of catastrophic forgetting which is known to affect the learning paradigms mentioned above. Finally, we propose a new adaptive computational model to some extent inspired by the “Monitoring and Adjustment” theory: the Speaker keeps track in her memory of the Listener's mistakes and, in later turns, it generates a caption that avoids such misunderstandings. The contrast set is not co-present from the start, as in Hawkins et al. (2020a), but it becomes partially available through interaction, i.e., through the images wrongly selected by the Listener.

### 2.2. Pragmatic speaker

The dynamic adaptation process at the core of the studies described above is strongly related to the Rational Speech Act (RSA) framework introduced in Frank and Goodman (2012). RSA model implements a social cognition approach to language aiming to capture the idea that speakers produce informative utterances (Grice, 1975). Moreover, it builds on the assumption that the meaning of a linguistic expression heavily depends on the context in which the expression is used, and hence it needs to be inferred. In this framework, speakers and listeners reason about each other's vision and exploit prior beliefs to interpret or generate an utterance. By doing so, the Speaker generates pragmatically informative descriptions. Intuitively, the RSA speaker achieves this ability by reasoning not only about what is true but also about the Listener's reasoning. For instance, in the context of a visually grounded referential game in which the Speaker has to describe an image and the Listener has to identify it within a set of images, the Speaker could assign equal probability to a variety of descriptions, but it will base its choice about what to generate by reasoning about which utterance will help the Listener identify the target the most. It does so by starting as a literal speaker that generates a number of captions; simulating a rational listener that reasons about the chosen utterance and picks the image that best matches such description; and finally acting as a rational speaker who takes such simulated choices into account and utters the most informative caption. The reader is referred to Goodman and Frank (2016); Franke and Jaeger (2016) for further details on the RSA framework and to Hawkins et al. (2022) for a very recent extension based on continual learning.

The RSA model has been implemented for the first time as a statistical classifier in Monroe and Potts (2015). The importance of combining pragmatic reasoning with neural networks was advocated in Andreas and Klein (2016) and has more recently been exploited within IC Generation (Schüz et al., 2021) system. Interestingly, Zarrieß and Schlangen (2019) combine RSA with a zero-shot setting to generate referring expressions describing objects whose categories are unknown to the Speaker. Similar to this last work, our RSA based model has to learn to refer to unknown object categories, but in our case the categories are unknown to the Listener and known to the Speaker. Bao et al. (2022) extend RSA to accommodate the Speaker-Listener's vocabulary disparity through Reinforcement Learning. Differently from them, we exploit RSA to help generate discriminative captions that are then promoted by the likelihood loss if they lead to a successful interaction. We rely on the incremental RSA technique proposed in Cohn-Gordon et al. (2018) in order to generate pragmatically informative descriptions about a target in a given set of images. Earlier RSA implementations sampled from the space of all the possible complete utterances; to this end, they use only a small part of the utterance space, leading to captions that were not always the most pragmatically informative. To overcome this limitation an incremental approach has been proposed. performing an inference for each step of the utterance generation. This step could be implemented both generating the sentence word-by-word (Cohn-Gordon et al., 2019) or can be defined in terms of different linguistic units, such as characters (Cohn-Gordon et al., 2018). The latter is the most efficient approach, since the former would require sampling from about 20,000 words, while the latter would require sampling from about 30 characters at each step. Hence, in this paper, we use the character-based decoder of Cohn-Gordon et al. (2018) to model the Speaker pragmatic reasoning.

This technique supports the efficient generation of pragmatic descriptions leveraging RSA during the generation process operating through a character-level decoder. The advantage of using a character-level decoder over a word-level one is that, at each generation step, the action space is much smaller for the former, making the RSA technique we are using in the adaptation phase more effective and efficient.

## 3. Datasets and task

### 3.1. Datasets

Our agents will first acquire grounded conceptual knowledge through the IC pre-training phase; then the Speaker learns to adapt to the Listener by playing an iterative reference game. Below we describe the datasets we used for these two phases.

#### 3.1.1. Pre-training data

We want to evaluate the interaction between a domain expert, who knows all concepts of a certain domain, and a layman, who has partial knowledge of it. In order to instill different background knowledge in the Speaker and the Listener, the two agents were pre-trained on the IC task using different datasets. The Speaker, who is taken to be the expert, is pre-trained on images from the whole MS-COCO training set, containing image caption pairs about objects organized in 12 super-categories. Hence, she is an expert on all MS-COCO domains (we consider each super-category to be a domain). The Listener, who is the layman, is pre-trained on a subset of such data; for each of the MS-COCO super-category, he does not see any instances of *N* of its categories (for instance, of the super-category “animal” he sees dogs and cats but he does not see any horse and cow, because he has always lived in a city). For the experiments we report in this paper, we have taken one category per each super-category to be unknown, and we do not consider the super-category “person” since it consists of only one category.^2^

#### 3.1.2. Adaptation data

Following Hawkins et al. (2020a), the adaptation data consists of contrastive sets of four images that we gather from MS-COCO validation set. Differently from Hawkins et al. (2020a), we built the contexts using the following criteria focused on the Speaker-Listener knowledge-disparity: (1) two of the four images in each context contain a visually salient object of a category unknown to the Listener; (2) the other two images contain only visually salient objects of the known categories of the same super-category of the unknown one;^3^ (3) the four images are visually similar.^4^ By applying these constraints, we built 1.956 contexts; out of them, for each of the 11 MS-COCO super-category, we randomly selected six contexts to run the adaptation phase. In the following, we refer to the unknown object categories as “unknown images” or “unknown objects”.

### 3.2. Repeated reference game

**Figure 2:**
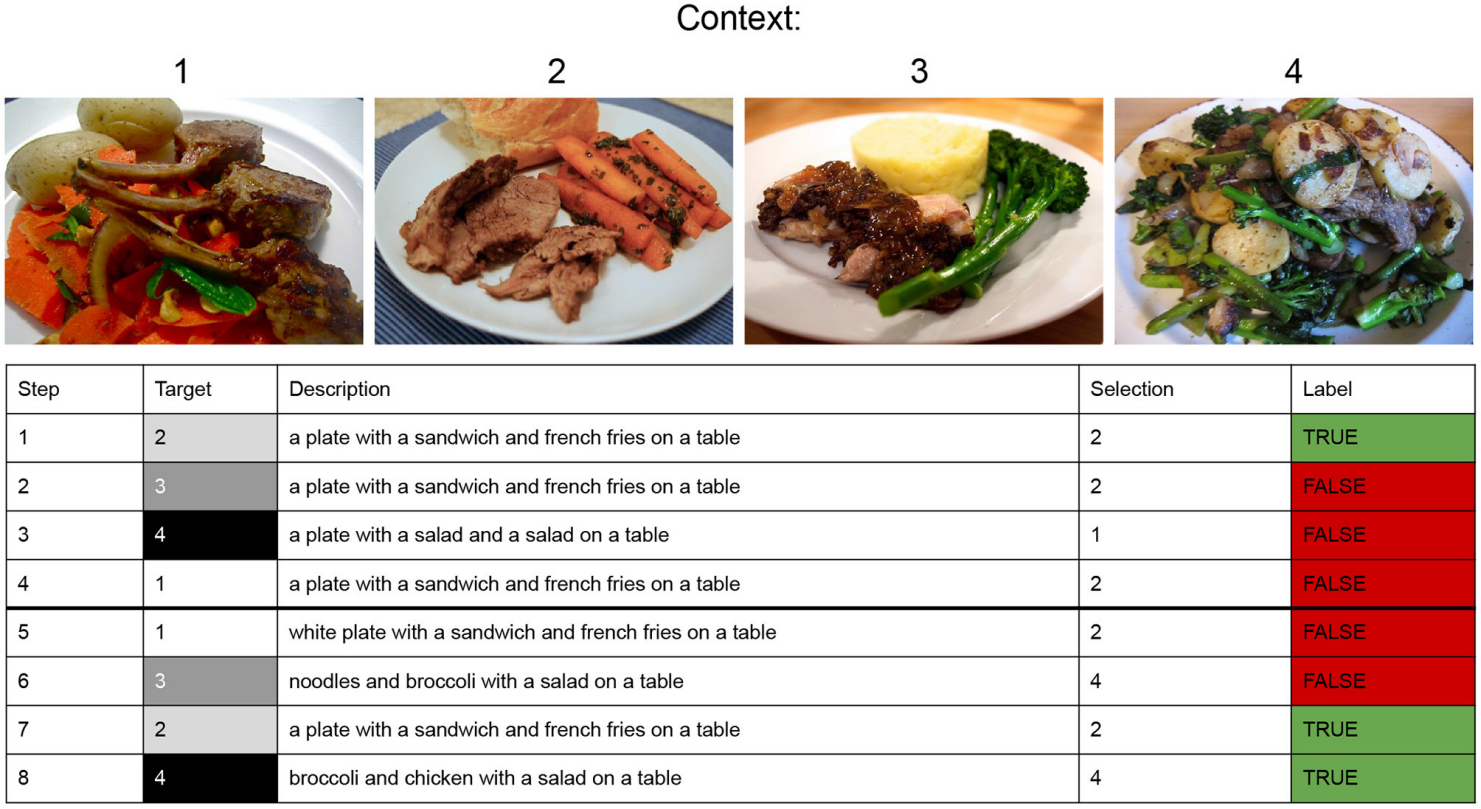
The Speaker is given a target image to be described; the Listener is given the 4 images (a context) among which he has to select the target. A turn consists of 4 exchanges (steps 1–4, 5–8, 9–12, 13–16, etc.), in each exchange the Speaker describes one of the 4 images of the context. Each context contains visually similar images with a salient object belonging to the same domain (same super-category, eg. food), two images contain an entity of a category unknown to the Listener (e.g., broccoli, image 3 and 4) and the other two are about known object categories (e.g., carrot, image 1 and 2). The adaptation phase runs for 10 turns. In the figure, only the first two turns are displayed.

We create iterative referential games each consisting of ten turns. As illustrated in Figure 2, each turn consists of four exchanges (steps). At each exchange, the Speaker receives one of the four images of a given context as target and has to describe it. The Listener receives the generated caption and has to select the target among the four images of the corresponding context. Within a turn, the order in which the target is given to the Speaker is random. Turn 1 consists of the first 4 steps. In the example game illustrated in the figure, in step 1, the Speaker is assigned to describe target 2. The Speaker generates a caption and the Listener correctly selects target 2. In step 2, the Speaker is assigned to describe target 3, the Listener receives the generated caption but wrongly selects target 2. The Speaker-Listener interaction continues with steps 3 and 4 in which the other images (4 and 1, respectively) are given as target. Turn 1 is therefore over and the next turn starts. Crucially, the interaction is about the same images which again are assigned randomly to the Speaker as target. Overall, after turn 2, the game goes on iteratively for other 8 turns during which the Speaker is asked to describe again the 4 images seen during the previous 2 turns.

Our main focus is to study whether and how models adapt their language to the Listener's knowledge within one iterative reference game. Differently from Hawkins et al. (2020a), who evaluate how a model adapts to human listeners, in our experiments the interaction happens between two models.

## 4. Models

We work with a Speaker and a Listener playing a reference game. For the Listener, we use the model used in Hawkins et al. (2020a) based on the standard encoder-decoder architecture (Sutskever et al., 2014). Given a context with four candidate images and a caption, the Listener selects what he thinks is the most plausible image the caption is talking about: for each of the four images, he computes the overall likelihood of the received caption out of the likelihood of the single words in the caption, and selects the image bringing to the highest likelihood. For the Speaker, we use the models described below.

### 4.1. Baseline

As the Listener, the Speaker is composed of an encoder representing the image through a ResNet-152 CNN and a decoder based on a Recurrent Neural Network with Long Short-Term Memory cells (Hochreiter and Schmidhuber, 1997) receiving the encoding of the image and generating a description of its visual content. As in Cohn-Gordon et al. (2018) and differently from Hawkins et al. (2020a), the decoder of the Speaker performs character-level modeling, predicting each character given the sequence of the previous ones. Given an image, the Speaker generates its description by encoding the image and selecting, at each step, the character having the highest value in the probability distribution computed by the decoder. We evaluate this model with no adaptation through the turns (Fixed model) and when updating its parameters at each successful exchange (LH model). In particular, when at time step *t* the Listener selects the correct target *I* after receiving the description *D*_*t*_ as input, the parameters θ_*S*_*t*__ of the Speaker are updated maximizing the likelihood of the description given the target image; the likelihood is defined as log*P*_θ__*S*__*t*___(*D*_*t*_|*I*). The procedure for the adaptation of the LH model is described as pseudocode by Algorithm 1.

**Algorithm 1:**
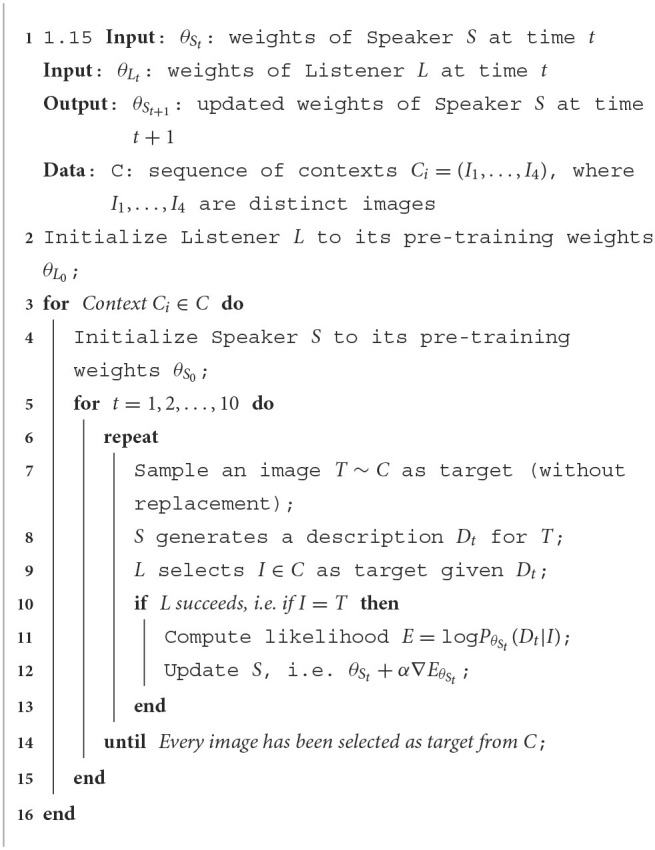
Adaptation procedure for the LH model.

### 4.2. RSA based models

The RSA based models rely on the implementation of the RSA framework proposed in Cohn-Gordon et al. (2018) who showed that the use of a character-based decoder makes the pragmatic inference manageable within a Language Model. In this incremental RSA implementation, the literal Listener generates a distribution over images based on the partial captions generated by the literal Speaker. The pragmatic speaker, given a target image, performs an inference over the set of possible characters to choose the one that will best lead the Listener to select the correct image. Building on such incremental RSA implementation, we propose RSA based models enhanced with a target-specific memory which works as follows. As with the baselines, given one of the 4 images of a certain context, the Speaker generates a description of it. The Listener receives the description and tries to guess which image in the context corresponds to the image described by the Speaker. Crucially, if the Listener selects a wrong image, the image is added to a target-specific memory used by the Speaker in the next turns. When the Speaker describes that target again, it will leverage RSA in order to find discriminative differences between that target and the image which has been mis-selected in the previous turn. To this end, the Speaker simulates the interaction using an *internal Listener*: among the possible true captions she could generate she selects the one which will let the internal listener distinguish the correct target from the other one. When the external Listener correctly guesses the target, the likelihood of the generated description given that target is increased and the parameters of both the encoder and the decoder are updated. Basically, RSA is used to guide the Speaker to generate descriptions that lead to successful exchanges based on the Listener's knowledge. Same as for the adaptation of the baseline, when at time step *t* the listener selects the correct target *I* after receiving the description *D*_*t*_ as input, the parameters θ_*S*_*t*__ of the Speaker are updated maximizing the likelihood of the description given the target image, defined as log*P*_θ__*S*__*t*___(*D*_*t*_|*I*). We will refer to this model as RSA LH.

In order to study the interplay between RSA and LH, on the one hand, we evaluate a Speaker that resets the target-specific memory when the Listener selects the correct image (RSA LH-reset), and on the other, we evaluate a model based only on RSA without LH, i.e., it does not increase the likelihood of the descriptions when the Listener guesses correctly. We will refer to this model as RSA. The procedure for the adaptation of RSA LH-reset is described as pseudocode by Algorithm 2. The RSA LH model follows the same pseudocode of RSA LH-reset, but it does not execute the instruction reported at line 16.

**Algorithm 2:**
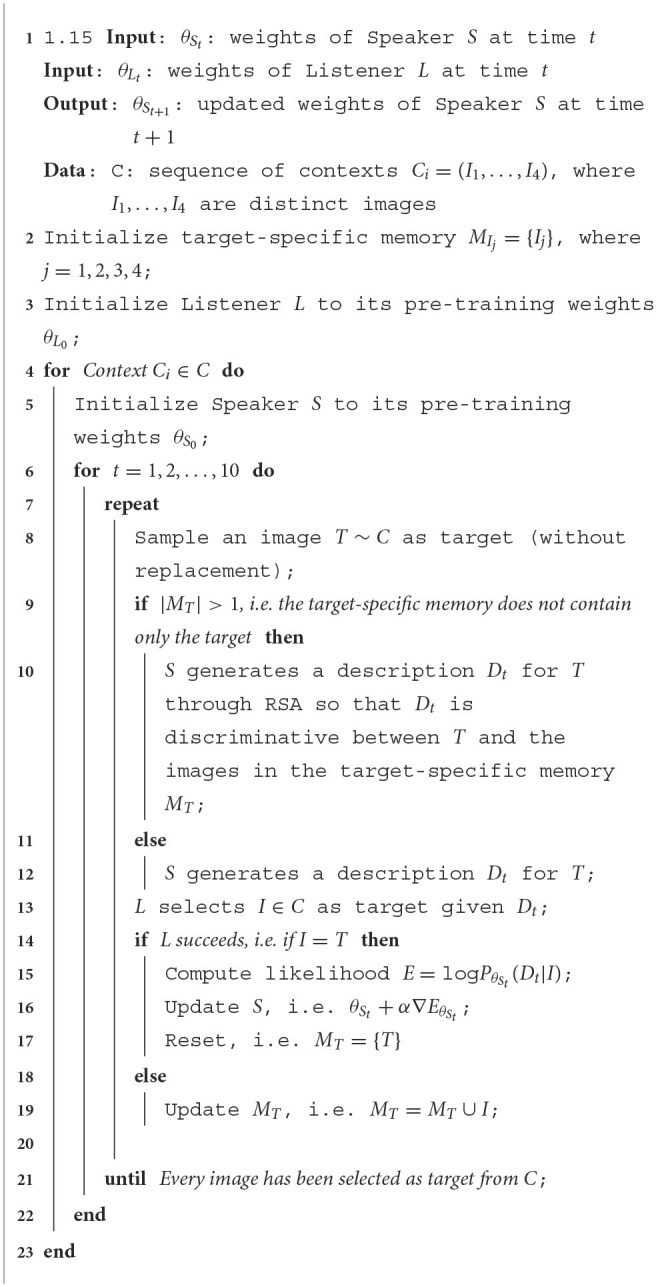
Adaptation procedure for the RSA LH-reset model.

## 5. Evaluation methodology

### 5.1. Experiments

We evaluate how models step-wise adapt to the Listener's knowledge when interacting iteratively on the same entities (as it is done in Hawkins et al., 2020a) (Experiment 1: Entity-Adaptation), and on any entity belonging to the same domain (Experiment 2: Domain-Adaptation). In the first case, we reset the models after each context, and hence evaluate how the models change through the ten turns of a certain context, while in the second case, we go one step higher, since we reset the models after each set of contexts within the same domain, and hence evaluate how the models change through them. To picture the impact of the Listener background knowledge on the Speaker's behavior, we will also evaluate models in a Peer-to-Peer setting, in which the Listener is a domain expert too, i.e., he has been pre-trained on IC using all the MS-COCO training set.

### 5.2. Metrics

Our aim is to understand whether the Expert successfully manages to communicate with the Layman. We measure this success in two ways: by evaluating how the Listener performs on the reference task (accuracy on the task success) and how the Speaker adapts her language use.

#### 5.2.1. Accuracy

We compute the average accuracy obtained by the Listener at each turn when the target image contains the unknown category vs. when the target image contains only known categories. In Experiment 1, we average on the turns involving the same context, instead in Experiment 2, we average across each turn of each of the six contexts, and report how the accuracy changes overall from the first to the last turn. Therefore, the accuracy per turn measures how much on average the Listener improves in guessing the target during the adaptation process. We interpret an increase in accuracy over turns as a sign that the descriptions of an object of an unknown category produced by the Speaker become more understandable to the Listener, in other words that the Speaker has adapted to the Listener.

#### 5.2.2. Language properties

Hawkins et al. (2020b) have shown that through repeated interactions with the same interlocutor, humans tend to shorten their utterances, drop closed-class parts of speech more than open-class parts of speech. On the contrary, in our knowledge disparity setting, we would expect the Speaker to produce a more informative, hence longer, description of the unknown object categories, since we expect her to use more visual attributes (adjectives) and potentially fewer nouns referring to unknown concepts. To check this hypothesis, we run the analysis described below.

#### 5.2.3. Length

We compare the length of captions referring to images having known vs. unknown categories. For each context, we calculate the length of captions within each turn by simply tackling the sum of the length of all captions generated in the steps with a known vs. unknown object as target image. We would expect the length of captions referring to objects unknown to the Listener to be higher.

#### 5.2.4. Percentage of adjectives

We measured the percentage of adjectives with respect to the total number of words occurring in the generated captions from the first turn to the last. We sum the total number of adjectives, tagged with NLTK (Bird et al., 2009), divide it by the sum of the length of captions, and multiply this value by 100. We do this for each turn and take the average across contexts.

#### 5.2.5. Unknown vs. known nouns

We measure the number of nouns used by the Speakers which refer to object categories that are unknown vs. known to the Listener. For each caption generated by the speaker for unknown (resp. known) images, we check if the caption has any mention of the unknown (resp. known) category. If it does, we calculate its frequency within the caption. We then compute the sum of all these frequencies and average it with respect to the length of captions to get the final percentage.

## 6. Results

### 6.1. Task success

**Figure 3:**
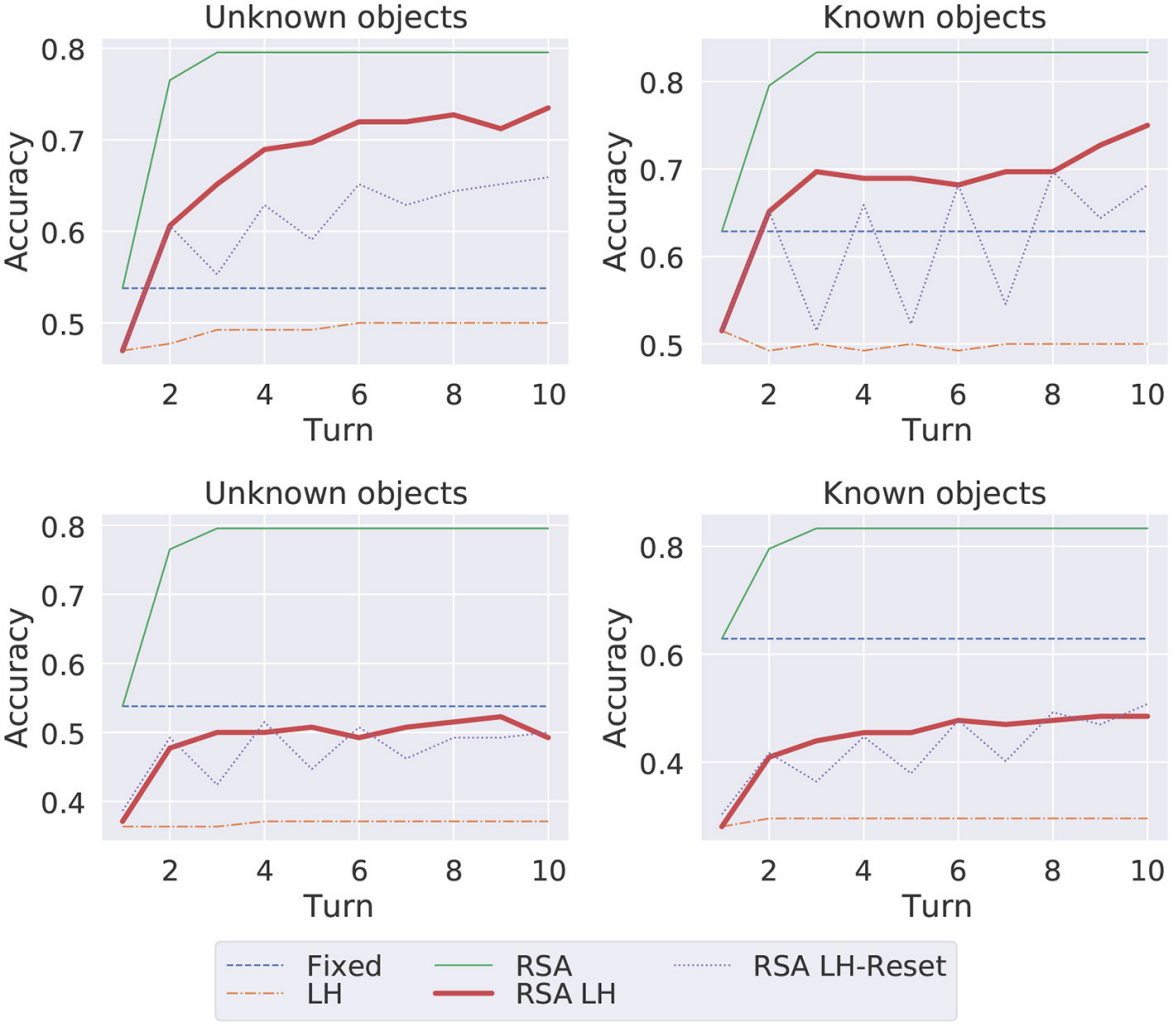
Average overall accuracy per turn (1st-10th) when the target image is an unknown vs. known object. Models' parameters reset after each context (Entity-adaptation, **Top**) vs. after each set of 6 within domain contexts (Domain-Adaptation, **Bottom**).

In Figure 3, we report the overall accuracy that models obtain through the adaptation process across the turns when their parameters are reset after each context (Experiment 1—plots in the upper part) or after each set of within-domain contexts (Experiment 2—plots in the bottom part). We compare the results obtained when the target image contains an unknown vs. known object category.

Obviously, communication is harder when the image contains an object category unknown to the Listener as shown by the lower accuracy the Listener obtains with the captions of unknown images produced by the Fixed model. Surprisingly, the LH model goes worst than the fixed ones in the known images, showing the negative impact of the adaptation based just on the likelihood loss even within the same turn. As expected, both for the unknown and known images the RSA is the best model and RSA LH is the model that, both for known and unknown images, goes closer to it. However, these two models have always access to the images mis-selected by the Listener in their target-specific memory. Therefore, computationally they are more costly and we would say also less cognitively plausible (they rely on a larger, and continuously growing, memory diary). The RSA LH-reset instead, once it has learned which caption is more suitable for the Listener, is able to utter such Listener-specific description even in the absence of the mis-selected image(s). Therefore, we believe its results are promising since they show a more adaptive consolidation behavior: it sees the mis-selected image only at the turn after the mistake is performed and in later turns it has to exploit the likelihood loss to produce a caption informative for the Listener. Its curve shows the model struggles to correct herself in the first five turns, but after such interaction succeeds in adapting to the Listener (from the 6th turn onward the performance of the Listener keeps on improving.

**Figure 4:**
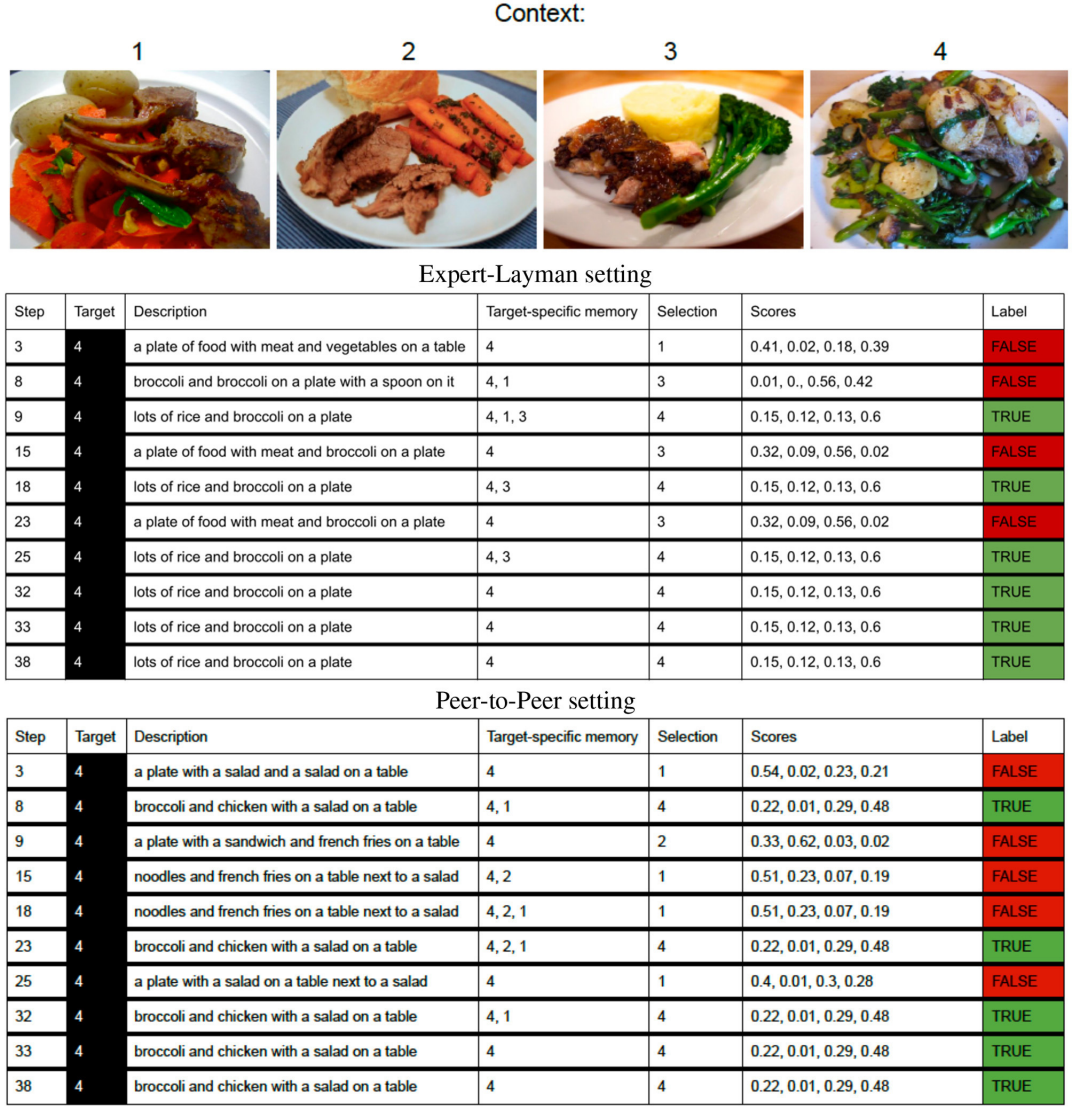
An example of interactions between the Speaker relying on the RSA LH-Reset model and the Layman **(Upper part)** and the Expert **(Bottom part)** Listener. For each of the ten turns, it shows the step in which the Speaker has to describe as target the image 4, which contains the concept of broccoli not known by the Layman Listener. When the Listener fails to select the target, at the next turn, at the step in which the Speaker has to identify that target again, she exploits her memory trace of the previous interaction: she aims to generate a caption that discriminates the target (image 4) from the mis-selected image. For instance, in the interaction with the Layman **(Upper part)**, in steps 3 and 8, at turn 1 and 2, respectively, the Listener makes a wrong selection; in turn 3 (step 9) the Speaker profits from her memory about the mistakes done by the Listener in the previous turns, and produces a caption more informative for the Listener that leads to his success). After some such memory-based success, the Speaker is able to be understood by the Listener even though it is not relying on the target-specific memory anymore (the memory contains only the target image—it has been reset).

Figure 4 (upper part) shows an example of interactions between the Speaker, relying on the RSA LH-Reset model, and the Layman Listener. For each of the ten turns, it shows the step in which the Speaker has to describe as target image 4, which contains the concept of broccoli not known by the Listener. At each step, given the generated description, the Listener computes the probability score of each image and selects as target the one with the highest score. Whenever the Listener fails to identify the correct target, the wrongly chosen image is added to the Speaker's target-specific memory and stays there till the Listener succeeds. When the Speaker in the subsequent turn will be assigned that target again, she will exploit such memory trace and aim to avoid the confusion previously experienced by the Listener by generating a caption that, she thinks will help be more informative for the Listener to distinguish the target from the wrongly chosen image. As we can see from the figure, having the memory trace at disposal helps the model generate a caption more informative for the Listener (steps 9, 18). Interestingly, from step 32 the Speaker is able to be understood by the Listener even though it is not relying on the target-specific memory anymore (the memory contains only the target image). Besides this promising behavior, the model has also some weaknesses that should be addressed in the future. As we can see from the sample interactions, its descriptions sometimes refer to objects not present in the image, such as “spoon”, “rice”, “sandwich”, etc. This happens even in the captions that result to be more informative for the Layman. It will have to be understood whether the model is undergoing to a form of language drift and how to reduce the presence of hallucinations during grounded language generation.

All the adaptive trained models go worse than the Fixed model when they are provided with batches of within-domain contexts and are reset after each domain (experiment 2). This result suggests the models undergo catastrophic forgetting. Hence, in the following, we will focus on the language changes in the captions generated by the Speaker across turns during the entity-based adaptation phase.

### 6.2. Comparison with expert listener (peer-to-peer)

**Figure 5:**
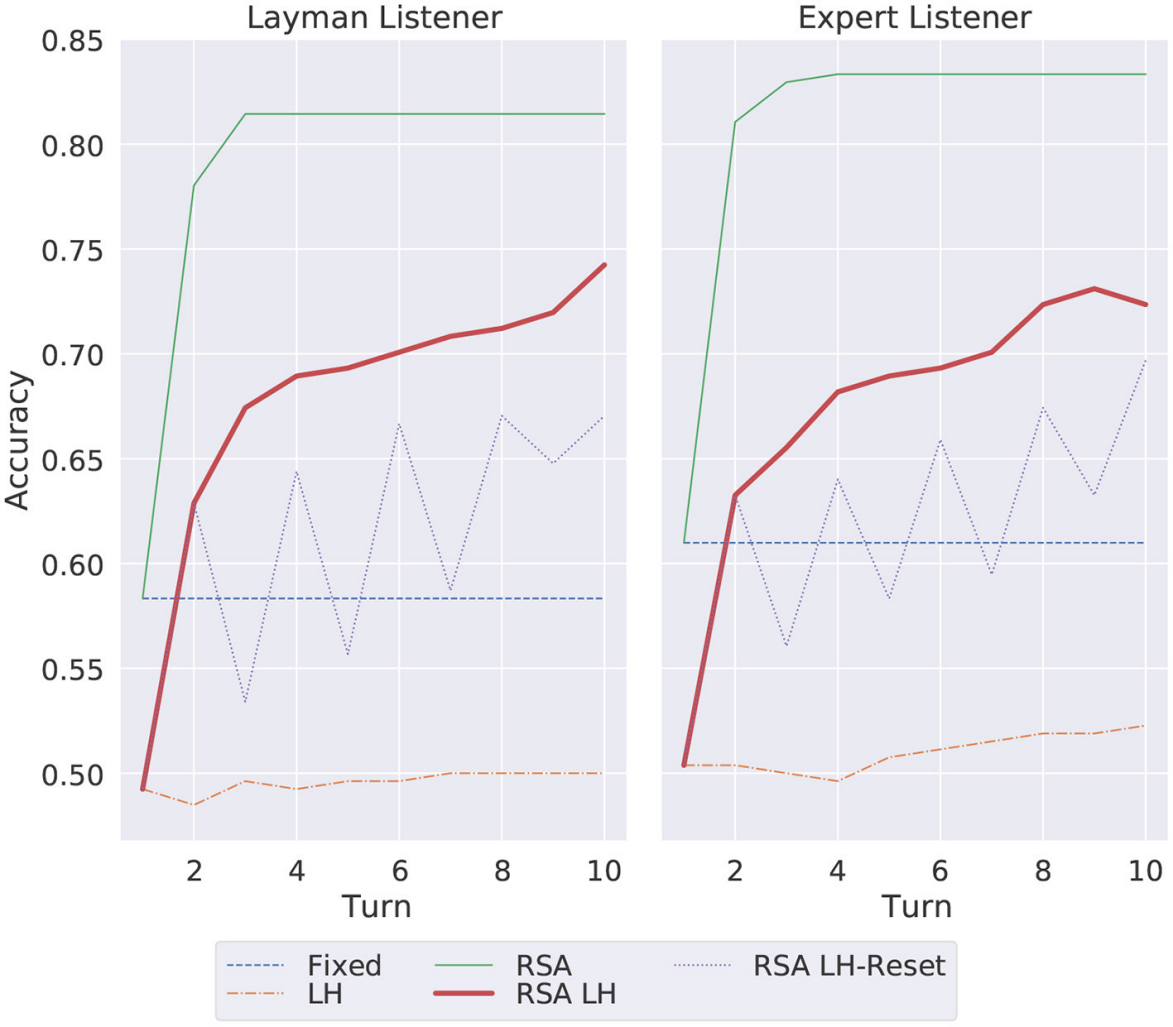
Average overall accuracy for all images per turn (1st-10th) when the Speaker communicates with the Expert vs. Layman Listener. The results have been computed at an Entity-adaptation level.

We experimented also with the peer-to-peer setting in which the repeated reference game happens between the Speaker and an Expert Listener trained on IC from the whole MS-COCO dataset. Recall that the Speaker and Listener do not share the very exact architecture: the former builds on a character-based decoder, the latter on a word-based one. Hence, the Listener is not expected to obtain 100% accuracy and the Speaker should still learn to adapt to her peer. Figure 5 shows the overall accuracy per turn (1st-10th) when the Speaker communicates with an Expert Listener and compares the results with the accuracy obtained by the Layman Listener discussed so far. These accuracies have been computed at the Entity-adaptation level. As expected, since the Expert Listener has experience, through the IC pre-training, of all object categories, his performance is higher than the Layman's one: the fixed model obtains around 62% (Expert) vs. 57% (Layman). The RSA puts a higher upper bound for the Expert Listener (around 84%), strangely the RSA LH seems to start decreasing its performance toward the end of the interaction, while the RSA LH-reset keeps on improving. The results confirm that the proposed RSA method is able to provide a more effective communication (i.e., leading to a higher accuracy) between the Speaker and the Listener even when there is no disparity between the knowledge of the two agents. Again, the results of the more efficient RSA LH reset model are promising. This happens because the target-specific memory allows the model to reason about the actual errors performed by the Listener in the context, allowing the Speaker to create new more effective descriptions which are more easily understood by the Listener, as illustrated by the bottom part of Figure 4.

### 6.3. Language use in the expert-Layman setting

**Figure 6:**
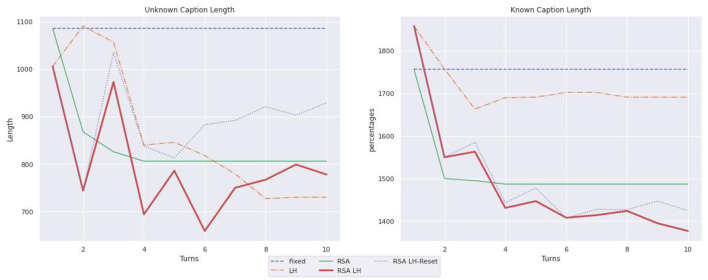
Length of captions referring to unknown vs. known objects.

**Figure 7:**
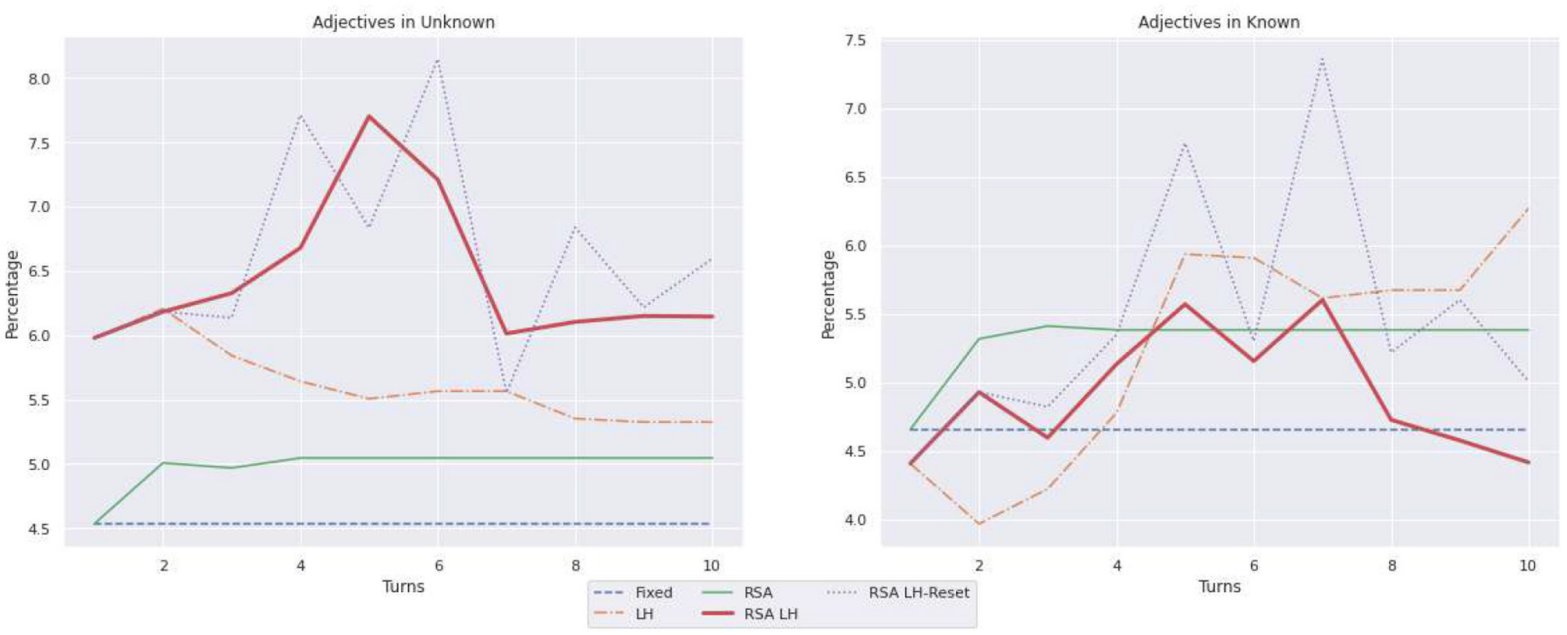
Percentages of adjectives, with respect to the total number of words, in captions describing unknown vs. known object categories.

**Figure 8:**
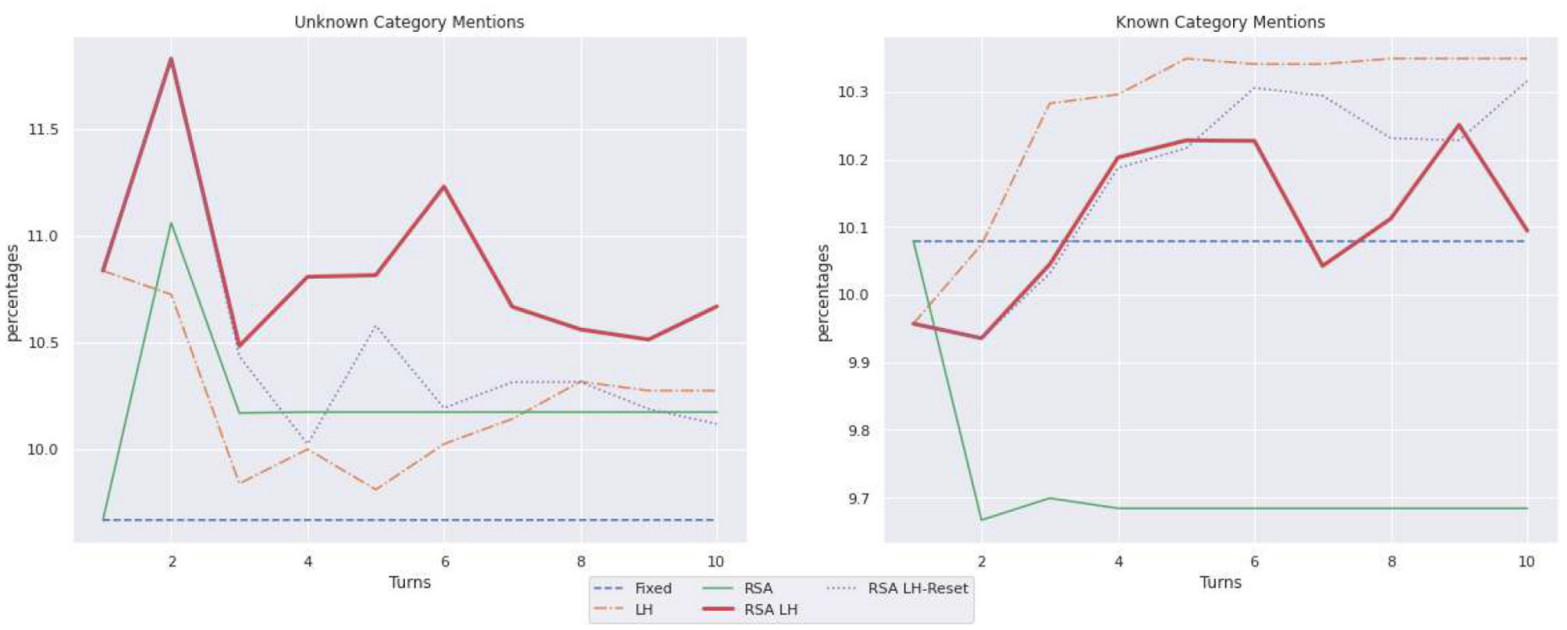
Average number of mentions of unknown vs. known object categories.

Table 1 summarizes the statistics illustrated by Figures 6–8 on the language use. It reports only the values for the last turn and, within parenthesis, the difference from the first to the last turn. As illustrated in Figure 6, when describing known objects, RSA LH and RSA LH-Reset are the models that shorten the caption length the most; for instance, the captions produced by RSA LH reset length decrease by 433 tokens, while the RSA model has a decrease of just 269 tokens. On the other hand, in the case of unknown objects, RSA LH and RSA LH-reset, after some shortening steps, learn to ask longer captions than LH in the last turns; RSA LH-reset is the model whose captions get longer in the last turn where it used 929 tokens, while e.g., LH uses just 730 tokens. In order to better understand the observed length changes, Figure 7 illustrates the use of adjectives. Moreover, RSA LH and RSA LH-reset use more adjectives when describing unknown than known objects (at turn 10, 6.14 vs. 4.41, and 6.59 vs. 5.01, resp.). Interestingly, they are also the models that increase the use of adjectives the most through interactions, as one would expect from an adaptive behavior. Finally, Figure 8 shows the average number of mentions of unknown vs. known object categories. All models refer to unknown categories less as the number of turns increases. On the other hand, mentions of known object categories increase for all models over time except for RSA, whose number of mentions to known object categories decreases during the first turns and then remains stable. This behavior might depend on the fact that RSA generates pragmatic descriptions which distinguish the target from the other objects in the contexts. Hence, it generates descriptions highlighting the differences between the target and the other images instead of directly referring to the target.

**Table 1:** Expert-Layman setting: language use at the last turn. Within parenthesis the language change from the first to the last turn (Turn 1 minus Turn 10).

	**Unknown categories**			**Known categories**		
	**Length**	**Adjectives**	**Cat. mentions**	**Length**	**Adjectives**	**Cat. mentions**
Fixed	1,060	4.53	9.67	1,756	4.65	10.08
LH	730 (280)	5.32 (0.66)	10.27 (0.56)	1,691 (167)	5.41 (−1)	10.35 (−0.39)
RSA	806 (280)	5.05 (−0.52)	10.17 (−0.5)	1487 (269)	5.38 (−0.73)	10.17 (−0.49)
RSA LH	778 (228)	6.14 (−0.17)	10.67 (0.16)	1,377 (481)	4.41 (−0.01)	10.09 (−0.12)
RSA LH reset	929 (77)	6.59 (−0.61)	10.12 (0.71)	1,425 (433)	5.01 (−0.61)	10.31 (−0.36)

### 6.4. Confusion between unknown vs. known objects

**Figure 9:**
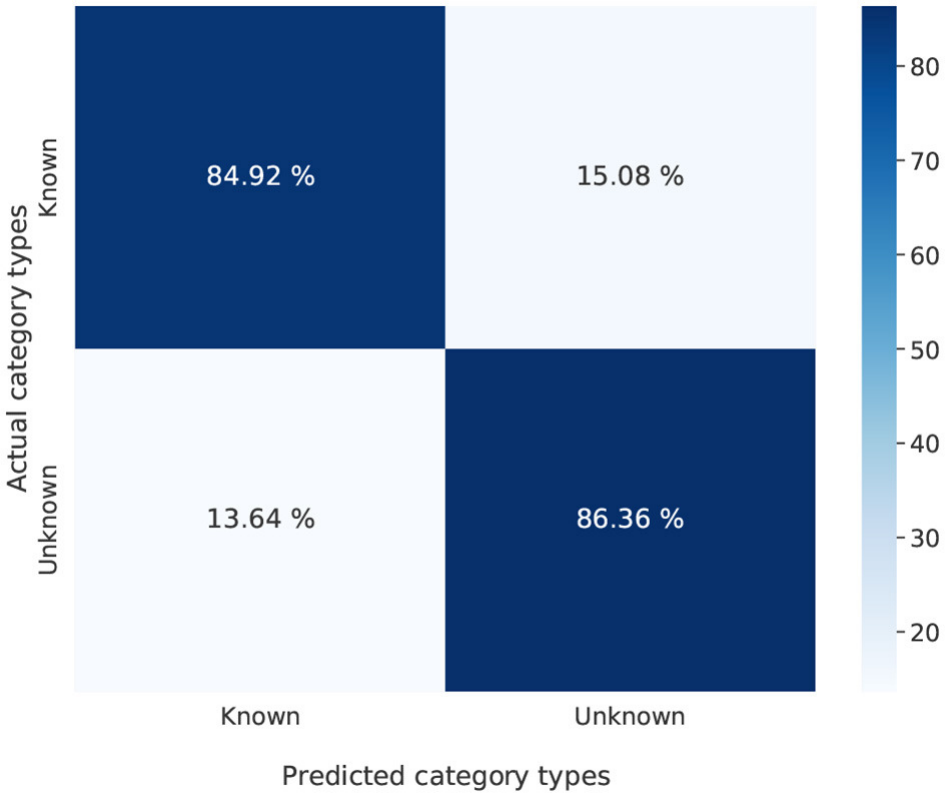
Confusion between unknown vs. known object category performed by the Listener when interacting with RSA LH.

In order to assess the kind of mistakes performed by the Listener when guessing the target image, we computed a confusion matrix reporting, for target images containing unknown vs. known objects, the number of predictions referring to each of the two kinds of objects. Figure 9 illustrates such confusion matrix for RSA LH; it shows that, when mis-selecting the candidate image, the Listener confuses an unknown (respectively, known) object with another unknown (respectively, known) object. The same pattern has been noticed in all the other models, assessing that in general the Listener rarely confuses known objects with unknown ones. Hence, at the category level, the selection is appropriate.

## 7. Conclusion

With this work, we aimed to bring attention to an important aspect of communication, namely its interactive dynamic flavor through which interlocutors adapt to each other. We believe the time is ripe to face this challenge with neural network based natural language generation systems. In particular, we believe the progress on continual learning should be merged with frameworks based on pragmatic reasoning and the iterative settings, such as those used in Hawkins et al. (2020a) and Zhu et al. (2021), are a promising challenge to study adaptive models. We put the attention on language adaptation in an Expert-Layman interaction where the Expert needs to adapt to the Layman's lack of domain knowledge.

We studied the captions generated by a Speaker who exploits the Listener's mistakes to produce more discriminative captions and learn from his success to become more informative for her interlocutor. We achieved this by relying on the RSA framework proposed in Frank and Goodman (2012) and by integrating it with the neural IC model described in Cohn-Gordon et al. (2018); the RSA step prompts the model to produce captions that discriminate a target image from the one mis-selected by the Listener in a previous interaction, and promotes such discriminative caption through the likelihood loss. Our results show that RSA could indeed work as a backbone to drive the Speaker toward the Listener: the communication becomes more successful (as shown by the increased accuracy) and the language generated by the Speaker features changes that signal an adaptation to the Listener's background knowledge. In the following we highlight the limitation of the current work as well as the future directions it opens.

## 8. Limits and future directions

The work we have reported has some limitations that could be addressed in the future. First of all, the character based decoder we have used, though makes computation efficient, generates captions with hallucinations; as the mention of e.g., “sandwich” in the example reported in Figure 4 and such hallucinations are present even in what is expected to be the result of the adaptive process. Some recent findings on decoding strategies have shown that hallucinations could be reduced by linking the decoder with a “reasoning module”, for instance the Guesser module in Testoni and Bernardi (2021), we hope further work will be done in this direction also with the character-based efficient decoder. Moreover, we would like to see a detailed comparison between the character vs. word based decoder with respect to this issue. Moreover, we show that the models undergo catastrophic forgetting, this is an important problem that should not be left aside since the ability to interact with multiple agents is a core aspect of adaptation. Finally, while writing the paper, we found Bao et al. (2022) who also rely on a memory, but paired with Reinforcement Learning, in order to perform adaptation; it would be interesting to compare our proposed model with their work in order to evaluate differences arising in terms of both accuracy and linguistic adaptation during the communication between the two agents. While developing adaptive models and comparing the various technical scenarios, an interesting view to keep in mind is the cognitive plausibility of the models. This view could help focus on the adaptation process that is also computationally less costly and hence interesting for large-scale applications.

Our work addresses some interesting questions that could be further explored and opens new challenges. First of all, our model receives the image mis-selected by the Listener and exploits it to produce a more discriminative and hence more informative description. It would be interesting to study whether the Speaker could learn to predict the mistake of the Listener, and produce an informative description based on such prediction. Moreover, the Speaker should be challenged to interact back and forth with Listeners with different knowledge, and still be able to produce tailored descriptions. A possible solution to address this limitation could come by using in the RSA based models an internal listener for each external ones so as to build and maintain a Listener-specific representation. Furthermore, the Listener should learn from the interaction with the Speaker to build representations of the unknown categories. Benotti and Blackburn (2021) call for collaborative grounding to negotiate meaning by collaborative recovery from the mistakes. In a similar spirit, convincing evidence has been brought from neuroscientists. Hasson et al. (2012) and Nguyena et al. (2019) trace the reason of linguistic alignments, noticed by psycholinguists (Garrod and Pickering, 2004), into brain alignment; in other words the speaker's brain responses during speech production are coupled with the listener's brain responses during speech comprehension. It would be interesting to study neural networks alignment. In this view, it would be interesting to train the two systems jointly so as to let them align and revise their conceptual knowledge cooperatively.

Understanding how to learn and adapt to different users with their own preferences is important for future development of many applications, such as home robots, voice assistants, and virtual online shopping, where each customer has their own way of communicating. With the current development of many smart home devices and virtual assistants, it is time to bring them to the next level, where their tasks are not only limited to understanding customers' requests, but also to learning and adapting to context, and combining knowledge coming from different sources, e.g., from language communication as well as visual perspectives to solve more complex tasks. Our work is going in the direction of enabling adaptation to customers and building the common knowledge to understand their preferences better.

## Data availability statement

The original contributions presented in the study are included in the article/supplementary material, further inquiries can be directed to the corresponding author.

## Author contributions

CG implemented the models. DB built the dataset and evaluated the language use. RB designed the project and lead it. D-TL gave feedback with a special eye to the application. All authors contributed to the paper writing. All authors contributed to the article and approved the submitted version.

## Footnotes

^1^The dataset and code can be found here: https://github.com/claudiogreco/frontiers2023.

^2^The unknown categories are: “laptop”, “microwave”, “bowl”, “umbrella”, “cow”, “tennis racket”, “broccoli”, “clock”, “potted plant”, “airplane”, “stop sign”.

^3^An object is visually salient if the total area occupied by all its instances in the image divided by the total area of the image is greater than or equal to 0.15.

^4^The similarity between images was computed as the cosine similarity between the feature vectors of images extracted from the final fully-connected layer of a ResNet-152 Convolutional Neural Network (CNN) (He et al., 2016) using the following steps: (i) we compute two sets of images containing unknown vs. known categories; (ii) we selected the top most similar unknown-known image pairs; (iii) from the set of images containing unknown (resp. known) categories, we retrieve the images most similar to the selected ones of the corresponding type—known vs. unknown.
